# Measures of infection prevention and incidence of SARS-CoV-2 infections in cancer patients undergoing radiotherapy in Germany, Austria and Switzerland

**DOI:** 10.1007/s00066-020-01681-1

**Published:** 2020-09-10

**Authors:** Christiane Matuschek, Johannes C. Fischer, Stephanie E. Combs, Rainer Fietkau, Stefanie Corradini, Kurt Zänker, Edwin Bölke, Freddy-Joel Djiepmo-Njanang, Balint Tamaskovics, Joachim E. Fischer, Martin Stuschke, Christoph Pöttgen, Robert Förster, Daniel R. Zwahlen, Alexandros Papachristofilou, Ute Ganswindt, Rainer Pelka, E. Marion Schneider, Torsten Feldt, Björn Erik Ole Jensen, Dieter Häussinger, Wolfram Trudo Knoefel, Detlef Kindgen-Milles, Alessia Pedoto, Olaf Grebe, Martijn van Griensven, Wilfried Budach, Jan Haussmann

**Affiliations:** 1 Medical Faculty, Department of Radiation Oncology, Heinrich Heine University, Duesseldorf, Germany; 2 Institute for Transplant Diagnostics and Cell Therapeutics, Heinrich Heine University, Düsseldorf, Germany; 3 Department of Radiation Oncology, Technical University of Munich, Munich, Germany; 4 Institute of Radiation Medicine (IRM), Helmholtz Zentrum Munich, Munich, Germany; 5 Partner Site Munich, Deutsches Konsortium für Translationale Krebsforschung (DKTK), Munich, Germany; 6 Department of Radiation Oncology, University of Erlangen, Erlangen, Germany; 7 Department of Radiation Oncology, University Hospital, LMU Munich, Munich, Germany; 8 University Witten/Herdecke, Witten, Germany; 9 Public Health, University of Mannheim, Mannheim, Germany; 10 Department of Radiation Oncology, University Essen, Essen, Germany; 11 Institute of Radiation Oncology, Cantonal Hospital Winterthur, University of Zurich, Zurich, Switzerland; 12 Department of Radiation Oncology, University Hospital Basel, Basel, Switzerland; 13 Department of Radiation Oncology, Medical University Innsbruck, Innsbruck, Austria; 14 Institute for Applied Statistics, Unterföhring/Munich, Germany; 15 Division of Experimental Anesthesiology, University Hospital Ulm, Ulm, Germany; 16 Department of Gastroenterology, Hepatology and Infectious Diseases, Heinrich Heine University, Duesseldorf, Germany; 17 Department for General Visceral and Pediatric Surgery, Heinrich Heine University, Dusseldorf, Germany; 18 Department of Intensive Care Medicine, Heinrich Heine University, Dusseldorf, Germany; 19 Department of Anesthesiology, Memorial Sloan Kettering Cancer Center, New York, USA; 20 Department for Cardiology, Rhythmology and Intensive Care Medicine, Evangelic Hospital, Dusseldorf, Germany; 21 MERLN Institute for Technology-Inspired Regenerative Medicine, Department cBITE, Maastricht University, Maastricht, The Netherlands

**Keywords:** COVID-19, Oncology, Radiation oncoloy, Pandemic, Patient care

## Abstract

**Purpose:**

COVID-19 infection has manifested as a major threat to both patients and healthcare providers around the world. Radiation oncology institutions (ROI) deliver a major component of cancer treatment, with protocols that might span over several weeks, with the result of increasing susceptibility to COVID-19 infection and presenting with a more severe clinical course when compared with the general population. The aim of this manuscript is to investigate the impact of ROI protocols and performance on daily practice in the high-risk cancer patients during this pandemic.

**Methods:**

We addressed the incidence of positive COVID-19 cases in both patients and health care workers (HCW), in addition to the protective measures adopted in ROIs in Germany, Austria and Switzerland using a specific questionnaire.

**Results:**

The results of the questionnaire showed that a noteworthy number of ROIs were able to complete treatment in SARS-CoV‑2 positive cancer patients, with only a short interruption. The ROIs reported a significant decrease in patient volume that was not impacted by the circumambient disease incidence, the type of ROI or the occurrence of positive cases. Of the ROIs 16.5% also reported infected HCWs. About half of the ROIs (50.5%) adopted a screening program for patients whereas only 23.3% also screened their HCWs. The range of protective measures included the creation of working groups, instituting home office work and protection with face masks.

Regarding the therapeutic options offered, curative procedures were performed with either unchanged or moderately decreased schedules, whereas palliative or benign radiotherapy procedures were more often shortened. Most ROIs postponed or cancelled radiation treatment for benign indications (88.1%). The occurrence of SARS-CoV‑2 infections did not affect the treatment options for curative procedures. Non-university-based ROIs seemed to be more willing to change their treatment options for curative and palliative cases than university-based ROIs.

**Conclusion:**

Most ROIs reported a deep impact of SARS-CoV‑2 infections on their work routine. Modification and prioritization of treatment regimens and the application of protective measures preserved a well-functioning radiation oncology service and patient care.

**Electronic supplementary material:**

The online version of this article (10.1007/s00066-020-01681-1) contains supplementary material, which is available to authorized users.

## Introduction

The coronavirus disease 2019 (COVID-19) outbreak has become one of the greatest challenges for modern societies, economies and medicine. By now, most countries in the world have been affected by this pandemic viral infection [1–7].

The SARS-CoV‑2 infection poses a significant threat to cancer patients as they can be more susceptible to the pathogenic complications associated with the infection compared to the general population [2]. Patients with malignant tumors are generally older and affected by additional comorbidities [8]. Furthermore, oncological treatments including chemotherapy, radiotherapy and the use of additional systemic agents might be associated with lymphopenia and result in an impaired immune response to viral infections [9]. Additionally, cancer fever and other nonspecific symptoms may mask the signs of early COVID-19 infections.

Early reports from China suggested an increased incidence of COVID-19 in patients with active cancer and major complications (such as the need for mechanical ventilation or death) might be 3–4 times higher in this population [10]. Reports from Italy confirmed that at least 16.5% of all deceased cases had in retrospect a history of cancer within the last 5 years [11].

Radiation oncology institutes (ROIs) present a unique challenge at the present time. Immune compromised patients with life-threatening diseases must visit these facilities daily to receive their treatment, being exposed to caregivers and fellow patients who can be asymptomatic carriers of the disease. Caregivers including radiation therapy technicians (RTTs), nurses, physicians and physicists have a higher incidence of exposure to the virus and contribute to further spreading the disease among coworkers and families. The RTTs generally meet about 30–50 patients per linear accelerator per day.

This work aims to quantify the incidence of COVID-19 in these departments, to measure and analyze the countermeasures taken by ROIs to decrease the risk of infection for both patients and healthcare workers (HCW), and to evaluate changes in treatment policy during the pandemic.

## Material and methods

### Questionnaire

We invited all registered ROIs of the German Society for Radiation Oncology (DEGRO) (*n* = 292) and the Austrian Society for Radiation Oncology (ÖGRO) (*n* = 16) to participate in an anonymous online survey on the COVID-19 outbreak and its clinical implications in their institutions. A link to the questionnaire was embedded in E‑mail messages sent between 26 and 27 April 2020. Additional invitations were sent on 5–7 May 2020 to all registered ROIs of the Swiss Society for Radiation Oncology (SRO, SSRO) (*n* = 37). The number of ROIs is based on the DIRAC database of the International Atomic Energy Agency (IAEA) [12].

The questionnaire was designed to capture the specific numbers of COVID-19 infections of both patients and caregivers and to report what measures were taken to prevent COVID-19 infections. The web-based online survey was conducted using the SurveyMonkey tool (SurveyMonkey.com LLC, San Mateo, CA, USA; http://www.surveymonkey.com). Overall, the questionnaire consisted of 25 questions: 6 addressing how many patients and staff were infected, 9 screening procedures as well as the use of personal protective equipment (PPE) and other prevention strategies, and 10 investigated changes in oncological treatments and follow-ups due to the pandemic. The characteristics of ROIs were investigated with four questions, and an open field one was placed at the end (Table 1). The questionnaire, provided in German and English, can be found in the Electronic Supplementary Material. The time to respond was set at 3 weeks. A high response rate made a reminder e‑mail unnecessary. The survey was closed on 22 May 2020.

**Table 1:** Structure of the online questionnaire

Section	Questions
Institution characteristics	Q1–Q3
Incidence and treatment of infected patients	Q4–Q6
Patient frequency (current vs. prepandemic)	Q7–Q8
Patient screening strategies	Q9–Q10
Staff screening, roster adjustments, personal protective equipment	Q11–Q19
Changes in oncological treatments and follow-ups due to pandemic	Q20–Q24
Open-ended response	Q25

### Statistical analysis

The datasheet from SurveyMonkey was adapted by eliminating double, truncated or implausible entries. For statistical analysis, ordinal entries were transformed to continuous scale values, e.g. for numbers of employees or change of policies “4–6” into mean value of the range (5). Changes in treatment and follow-up policy were factorized to sum 100%.

Several hypotheses were formulated prior to the data analysis. We hypothesized: 1) a decrease in the number of patients treated in ROIs during the pandemic, 2) the type of ROI and the presence of positive SARS-CoV‑2 results in the patients caused changes in treatment protocols, such as the use of fewer fractions, postponement or omission of radiation treatment (RT).

To clarify whether changes in radiation policy were contingent on the presence of the infection and the specific institution, the 19 variables describing this policy (curative, palliative, benign, chemotherapy and systemic therapy) were analyzed using principle component analysis for dimensionality reduction. Components with an eigenvalue >1 in all subgroups were included, resulting in 5 relevant components. The maximum distance of the factors was calculated. The highest 10% of the distances were further compared.

German COVID-19 incidences were taken from the Robert Koch Institute (RKI) accessed on 22 May 2020 [13]. The Austrian incidence of the infection was obtained from https://coronavirus.datenfakten.at/, while for the Swiss https://covid-19-schweiz.bagapps.ch/de-2.html was used, including the absolute numbers obtained as of 22 May 2020.

The pooled results of the questionnaire were reported in a descriptive form with total and relative numbers, means, and standard deviation. Statistical analysis was performed using Student’s t‑test for normally distributed data or by rank comparing or dichotomous statistics with *p*-values below 0.05 considered statistically significant.

Analysis was conducted in R (R core team, Auckland, New Zealand) and using IBM SPSS Statistics for Windows, Version 22.0. Released 2013 (IBM Corp, Armonk, NY, USA [14]).

## Results

### Baseline results

The questionnaire was open from 26 April until 22 May 2020. Nearly two thirds of the answers were provided within the first 2 days.

Tables 2, 3 and 4 show the number of responding institutions distributed by country and type of ROI. After exclusion of double entries, 106 answers to the questionnaire were analyzed, with 83, 13 and 10 responses from Germany, Austria and Switzerland, respectively. The response rates were 28.4% (83/292) for Germany, 81.2% (13/16) for Austria and 29.7% (11/37) for Switzerland. The largest number of responses (*n* = 31) came from ROIs in hospital departments, followed by ROIs within medical care centers (*n* = 28), ROIs in university clinics (*n* = 24) and private practices (*n* = 23).

**Table 2:** Baseline characteristics of patients and medical staff with the description of the COVID-19 positive cases in patients and HCWs

Baseline characteristics	*n*	
Overall	106	–
Germany	83	–
Austria	13	–
Switzerland	10	–
Practices	23	–
Medical care centers (MVZ)	28	–
Community hospitals	31	–
University clinics	24	–
Patients in ROIs	Mean	SD
Mean *n* patients currently	83.3	45.0
Mean *n* patients before pandemic	97.7	48.3

*ROI* radiation oncology institute

**Table 3:** Radiation treatment in SARS-CoV‑2 positive patients divided according to symptomatic status

SARS-CoV-2 + patients	*n*	
ROIs with positive patients	24	–
Positive patients	46	–
–	Asymptomatic/ low symptomatic	Symptomatic
RT stopped	2	5
RT break >1w	4	1
RT break < 1w	23	10
Other schedule	1	0

*w* week, *RT* radiotherapy, *n* number, *ROI* radiation oncology institute

**Table 4:** Baseline characteristics of patients and medical staff during the time of the first wave of the SARS-CoV 2 pandemic

Number of + HCW	0	1–3	4–6	7–9	*N* adjusted	%
ROI with + HCW	–	–	–	–	16	16.5
Physicians +	6	4	1	0	9	8.3
RTTs +	5	7	0	0	7	5.2
Physicists +	7	5	0	0	5	6.5
Nurses +	8	1	0	1	9	8.8
Administrative personnel +	7	3	0	0	3	6.0
Any HCW +	–	–	–	–	33	7.0

*ROI* radiation oncology institute, *HCW* health care workers, *RTT* radiation therapy technicians, *n* number

Of the 106 ROIs, 24 reported a total of 46 SARS-CoV‑2 positive patients. Notably, only two centers reported more than two infected patients (18 in one and 5 cases in the other) during radiotherapy. Most asymptomatic or oligosymptomatic patients (76.7%) continued their treatment with a break in RT of less than 1 week. Many centers with symptomatic patients stopped therapy or added a break for more than 1 week. Only 3 centers continued therapy in 10 patients either without a break or with 1 for less than 1 week. Most symptomatic cases continued their therapy (68.8%). The HCW tested positive in 16 ROIs (16.5%) with a similar distribution among the spectrum of roles.

As shown in Table 5 50.5% of the ROIs screened their patients for SARS-CoV‑2 with questionnaires being the most common test (46.9%). Taking the temperature was performed in 26.3% ROIs, while O_2_ saturation (2.0%), pulse rate (4.0%) and swab testing (9.1%) where done less frequently. A minority of ROIs (*n* = 20, 23.3%) performed screening for HCWs.

**Table 5:** Screening methods for patients and medical staff during the SARS-CoV‑2 pandemic

Patient screening					
*n* ROI screening patients	50	50.5%	–	–	–
**Frequency and type** **of Screens**	**Not Done**	**Weekly**	**More than Weekly**	–	–
Questionnaire	53	12	34	–	–
Temperature	73	2	24	–	–
Oxygen saturation	97	0	2	–	–
Heart rate measurement	95	1	3	–	–
Swab test	90	6	3	–	–
**HCW screening**	20	23.3%	–	–	–
**Number of screened** **HCW per week**	**0**	**0–10**	**11–20**	**21–50**	**51–100**
Symptoms (*n*)	6	6	4	2	2
Temperature (*n*)	12	5	0	2	1
Oxygen saturation (*n*)	18	1	0	0	0
Heart rate (*n*)	18	1	0	0	0
Nasopharyngeal swab (*n*)	7	8	3	0	2

*n* number, *ROI* radiation oncology institute, *HCW* health care workers

**Fig. 1:**
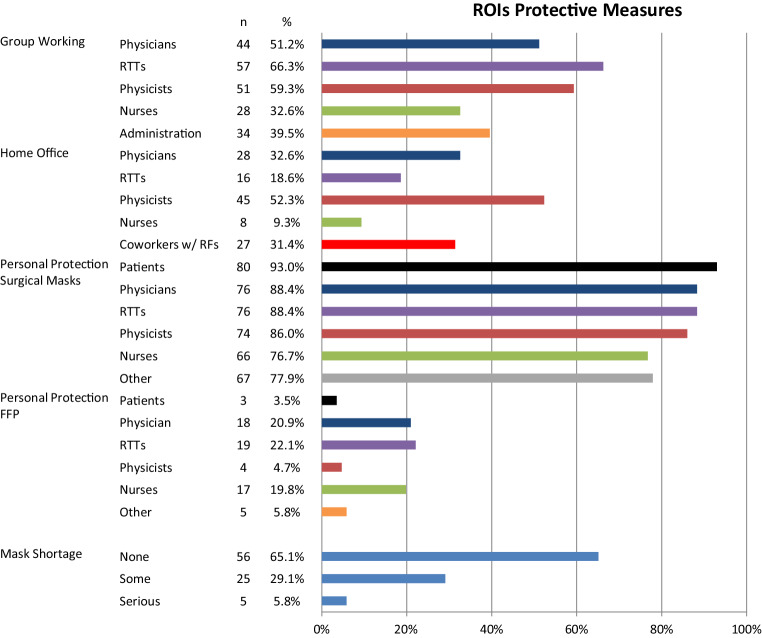
Overview of the implemented protective measures in all participating institutions for all workgroups

Fig. 1 assesses the use of protective measures for the ROI staff, including a rearrangement into work groups, work from home and the use of PPE. The majority of institutions reported smaller work groups for RTTs (66.3%), physicists (59.3%) and physicians (51.2%), whereas nurses (32.6%) mainly remained in the same teams. Working from home was more common for physicists (52.3%) and physicians (32.6%). Nurses (9.3%) and RTTs (18.3%) worked less frequently from home. In 31.4% of the centers, home office capabilities were organized for HCWs with an increased risk for severe clinical course of COVID-19, such as older age, lung disease or cardiac comorbidities.

Standard surgical masks were the main PPE used in all groups. A minority used FFP2/3 masks, most frequently for RTT (22.1%), nurses (19.8%) and physicians (20.9%). A temporary shortage of protective masks was reported by 34.9% of responding ROIs.

**Fig. 2:**
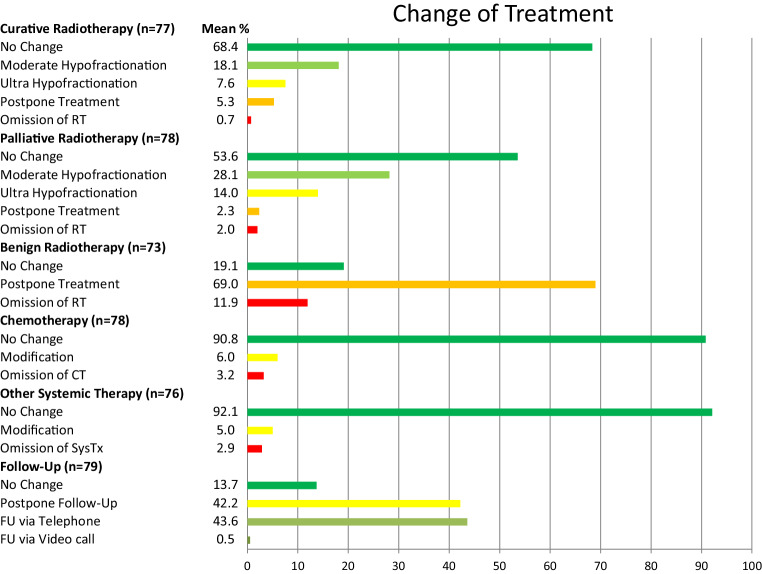
Type and frequency of change of therapeutic strategy and follow-up as reported by the ROIs

The question of how ROIs adapted their therapeutic regimens during the pandemic is addressed in Fig. 2. In patients with curative intentions, 68.4% of the centers did not alter the treatment plan. When RT schedules were changed, the centers usually adopted a moderate hypofractionation (18.1%) with little use of ultra-hypofractionation (7.6%) or postponement of therapy (5.3%). When treating patients with a palliative intent, more centers switched to shorter protocols (42.1%). Most of ROIs postponed or omitted RT for benign indications (88.1%).

Concurrent chemotherapy was administered as scheduled in 90.8% of cases. Some ROIs modified their usual strategies (6.0%) with omissions of chemotherapy in 3.2% of cases. Additional systemic treatments were administered as scheduled (92.1%), with some modifications (5.0%) or omissions (2.9%).

Follow-up visits were done via telephone (43.6%) or postponed (42.2%). Videochat was rarely offered within routine follow-up (0.5%).

### Statistical analysis

**Fig. 3:**
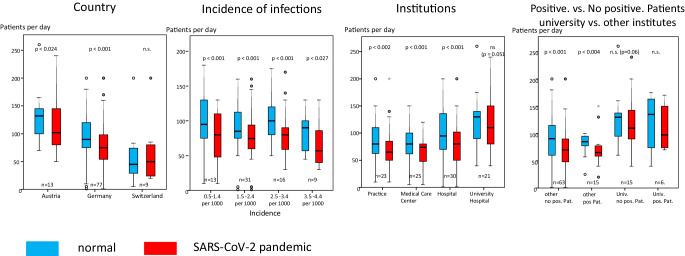
Comparison of mean number of patients treated per ROI per day before and during the pandemic separated by country, incidence, type of ROI and occurrence of positive cases. Bar denotes median, the box the first interquartile range (IQR, 50%), whiskers 1.5 IQR of the box. Outliers, when present, are marked as circles (more than 1.5 IQR out of the box) or as stars (more than 3 IQR out of the box)

Fig. 3 shows that Austrian (*n* = 13; *p* = 0.024) and German (*n* = 77; *p* < 0.001) ROIs treated significantly less cases after the beginning of the pandemic compared to before, whereas Swiss institutions did not report a reduction of case load (*n* = 9; *p* = n. s.). We further analyzed the impact of the SARS-CoV‑2 incidence per 1000 patients in the German institutions on change of patient cases per ROIs. As all comparisons showed significant reductions, the incidence did not seem to affect the caseload. The drop in patients also appeared to be independent of the type of ROI and whether or not the ROIs reported positive patients; however, the differences did not reach statistical difference for the university hospitals.

**Fig. 4:**
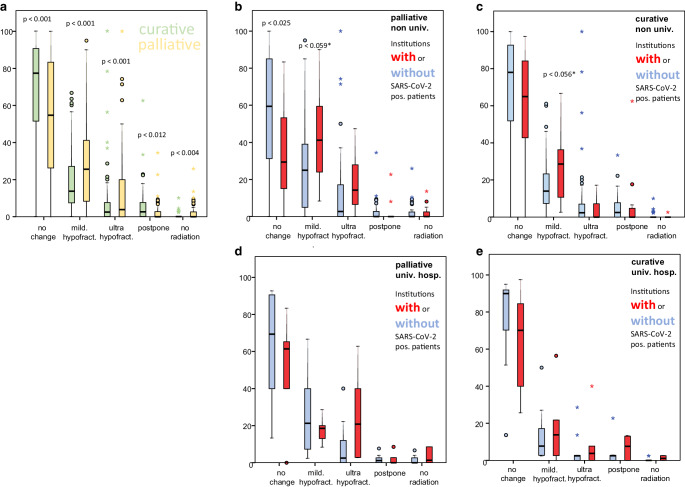
Analysis of changes in therapeutic concept: *left*: comparison of changes in therapy by curative and palliative treatment intention. Patients treated with a palliative intent were more likely to undergo changes in strategy than curative patients. *Right*: same analysis, divided by curative/palliative treatment intention, occurrence of positive patient cases and university and non-university clinics. Bar denotes median, the box the first interquartile range (IQR, 50%), whiskers 1.5 IQR of the box. Outliers, when present, are marked as circles (more than 1.5 IQR out of the box) or as stars (more than 3 IQR out of the box)

Fig. 4a–e shows the analysis of changes in therapeutic strategy to shorter treatment schedules, postponement or omission of radiation therapy by type of ROI and whether the institution reported positive patients.

We detected that curative schedules were more likely to stay unchanged, whereas moderate or ultra-hypofractionated treatment regimens were applied more in patients with palliative concepts. Postponing RT was more common in curative cases, in contrast to omission of RT in palliative situations. Moreover, we detected a tendency for non-university ROIs to change their RT-schedules compared to university clinics in curative and palliative cases.

**Fig. 5:**
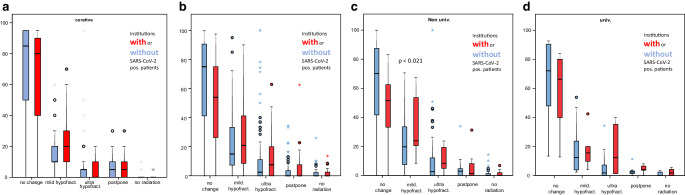
Strategy change in curative patients by occurrence of COVID-19 cases in the department. Shown are the mean number of ROIs reporting changes in their treatment concepts. **a** Treatment modalities for curative procedures, institutions with (*red*) or without (*blue*) SARS-CoV‑2 positive patients. **b** Change of treatment modalities for all patients institutions with (*red*) and without (*blue*) SARS-CoV‑2 patients. **c** Institutions with (*red*) or without (*blue*) SARS-CoV‑2 positive patients in non-university clinics. **d** Institutions with (*red*) or without (*blue*) SARS-CoV‑2 positive patients in university clinics

Fig. 5a–d demonstrated the changes in patients treated with a curative intend. Here the majority of ROIs reported mainly no changes or a switch to mild hypofractionation. The occurrence of SARS-CoV‑2 cases did not affect the change in patients treated with curative intent.

**Fig. 6:**
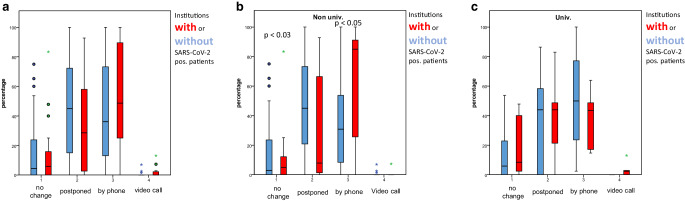
Changes in follow-up: mean number of changes to either no change, postponement of follow-up visit, switch to telephone call or video call. This was also separately analyzed for non-university and university clinics. **a** Changes in follow up, all institutions, Institutions with (*red columns*) or without (*blue columns*) SARS-CoV‑2 positive patients (univrsitary and non universitary clinics both together). **b** Changes of follow up, non-university clinics, Institutions with (*red columns*) or without (*blue columns*) SARS-CoV‑2 positive patients in non universitary clinics. **c** Changes of follow up, university clinics, Institutions with (*red columns*) or without (*blue columns*) SARS-CoV‑2 positive patients in universitary clinics

Fig. 6a–c shows the impacts on SARS-CoV2 on the follow-up concepts. Positive COVID-19 cases did not affect the changes in whole sample; however, non-university clinics with positive patients reported significantly more changes to follow-up visits by telephone.

## Discussion

The analysis of this online survey provides a cross-sectional assessment of ROIs in three European countries between the end of April and the end of May 2020, during the COVID-19 pandemic. The estimated cumulative COVID-19 incidence (0.56%) in ROIs is slightly higher than in the general population (0.25%), reflecting the presence of higher risk population in ROIs. Serious outbreaks in multiple ROIs have been avoided most likely because of the protective measures implemented by each institution in compliance with published reports and recommendations [15–21]. In March 2020, ARO, DEGRO and the professional association for radiation oncology during the COVID-19 pandemic (https://www.degro.org/stellungnahme-der-aro-degro-und-des-berufsverbandes-zur-strahlentherapie-waehrend-der-covid-19-pandemie) released a statement with suggestions on the treatment of patients suspected of SARS-CoV‑2 infection. These recommendations were in agreement with the current recommendations of the German RKI and local hygiene commissions.

These documents highlight the need to maintain the safety of both patients and HCWs by avoiding or rescheduling treatments if the risk of being infected with COVID-19 outweighs the benefit of treatment, and shortening therapies as much as possible. Proper PPE seems to reduce the transmission of the virus and protects HCWs [22, 23].

The American (ASTRO), European (ESTRO), Japanese (JASTRO) and the members of the Swiss (SRO SSRO) societies of radiation oncology published the results of similar surveys. Despite some regional differences, changes in clinical practice parallel the spread of COVID-19 in different countries. American centers appear to have implemented stricter screening procedures (98% screen patients and 91% HCW) compared to their European counterparts (82% screen patients and 60% HCW; our data: 51% screen patients and 23% HCW). Of the ASTRO centers 69% suffered from PPE shortage compared to 48% of ESTRO centers (our data 34.9%). The use of telemedicine was higher for ASTRO (89%) than ESTRO (72%) surveys. The number of patients wearing a mask differed between the surveys: ASTRO 83%, JASTRO 50.5%, SRO SSRO 59%, and our data 93%. Similarly, our results, a decline in referrals seemed to have affected institutions worldwide as 85% of American and 60% of the European centers reported reduced number of patients.

Kuderer et al. reported the largest analysis of cancer patients infected with SARS-CoV‑2 [24]. Fever, cough, fatigue/malaise and dyspnea were the most common symptoms and the crude death rate was estimated at 13%. Patients with active cancer had odds ratio 5.2 times higher for death compared to patients in remission or no evidence of disease. Concomitant systemic therapy, however, had no statistically significant influence on survival, non-cytotoxic (OR = 1.04) and cytotoxic effects (OR = 1.47), suggesting that omission of systemic therapy is probably unnecessary and should be strongly weighed against the potential benefits. Patients with solid tumors, often treated with radiation therapy, may be less at risk than patients with hematological malignancies. This finding is supported by Lee et al. who reported that patients undergoing any systemic anticancer treatment during the last 4 weeks including cytotoxic chemotherapy (OR = 1.18) and radiotherapy (OR = 0.65) had no increased risk of death according to an univariate and a multivariate analysis [25]. In contrast, Achard et al. reported an increased mortality after chemotherapy (OR 3.51) with no effect of radiotherapy [26].

Evidence for drastically higher mortality among SARS-CoV-2-positive cancer patients undergoing active therapy is controversial as data showing an increase are mostly retrospective and hampered by confounding factors. Specifically, radiation therapy as a cancer treatment modality is not linked to higher mortality from COVID-19; however, there is growing evidence on the additional harm from the COVID-19 infection in untreated malignant diseases with an increased morbidity and mortality [27, 28]. It appears to be critical for health care providers to maintain a functioning structure to treat other diseases. Especially, tumor-treatment often requires timely diagnosis and the participation of different medical departments.

Our survey shows that most of the curative treatment protocols were not affected by COVID-19, with infected cancer patients being able to continue their treatments, which demonstrated an intact infrastructure of the responding ROIs. This is in contrast with how other countries responded to similar scenarios. For example in Italy, in areas severely affected by the pandemic, the recommendations have been to postpone all non-urgent therapy and cancel palliative radiotherapy when other alternative protocols are equally effective [15]. Nonetheless, treatment of SARS-CoV‑2 patients has been proven to be challenging as well as time and resource intensive. Centers have to secure transportation of the infected patients to the ROIs, allow specific time slots on the machines and perform specific training to the treatment staff. Hygiene measures as well as constant ventilation of the treatment rooms have also to be considered.

Similar to our experience, the Swiss survey reported an increase of hypofractionation, even though with a lower percentage compared to ours (5–18% and 25.6–42.1%, respectively) [29]. It will be interesting to see whether ROIs will continue using the shorter treatment protocols, especially for curative treatments of breast or prostate cancers. In these sites, the use of moderate hypofractionated or ultra-hypofractionated regimens might still be lower compared to countries like the United Kingdom or Canada. One could speculate that some of these modifications in protocols will remain in practice as found to have improved patient outcome with less toxicity than anticipated.

We acknowledge several limitations of our survey. It reflects only a certain interval within the timeframe of the pandemic. The number of responding centers was limited, with a 31% response rate. This is comparable to 23.3% of the JASTRO survey [30]. Some parts of the raw data had to be transformed into continuous variables to allow statistical analysis, which might introduce bias. The number of COVID-19 cases is probably underreported as neither broader PCR nor antibody testing was performed at the time of the survey. Asymptomatic, yet infectious cases could have been present, but not detected [31, 32].

This survey might be considered as a starting point for future studies. Comparative studies (ROI vs. general population or ROI vs. other organizations with increased patient contact) would add additional medical information on this topic. Data could be collected on the medical and financial repercussions of prevention strategies in different ROIs, or at the regional or national level, on the assessment of risky behavior and their consequences, with the identification of the responsible factors.

## Conclusion

This survey demonstrated a significant effect of the COVID-19 pandemic on the responding ROIs, with implementation of safety measures and changes of their treatment protocols. The ROIs were able to perform curative treatments and persisted to mainly continue radiotherapy to SARS-CoV‑2 positive patients. The study also aims to raise awareness to new clinical needs to cope with COVID-19 infections in the future.

## Caption Electronic Supplementary Material

ESM1: Questionnaire on the situation of radiotherapy in Germany during the SARS-CoV-2 pandemic

## Abbreviations

ARO: Arbeitsgemeinschaft für Radiologische Onkologie = Working group for radiation oncology
ASTRO: American Society for Radiation Oncology
COVID-19: Coronavirus disease 2019
DEGRO: Deutsche Gesellschaft für Radioonkologie (German Society for Radiation Oncology)
DIRAC: Directory of Radiotherapy Centers
ESTRO: European Society of Radiation Oncology
FFP2/3: Filtering facepiece (mask) class 2/3
HCP: Healthcare professional
HCW: Healthcare workers
IAEA: International Atomic Energy Agency
JASTRO: Japanese Society of Radiation Oncology
MVZ: Medizinisches Versorgungszentrum (medical care center)
ÖGRO: Österreichische Gesellschaft für Radioonkologie, Radiobiologie und Medizinische Radiophysik (Austrian Society for Radiation Oncology, Radiation Biology and Medical Radiophysics)
PPE: Personal protective equipment
RKI: Robert Koch Institute
ROI: Radiation oncology institute
RT: Radiation therapy
RTT: Radiation therapy technician
SARS-CoV‑2: Severe acute respiratory syndrome coronavirus 2
SRO SSRO: Swiss Society for Radiation Oncology
