# Supplementary material for: Measures of infection prevention and incidence of SARS-CoV-2 infections in cancer patients undergoing radiotherapy in Germany, Austria and Switzerland
Source: Strahlenther Onkol. 2020 Sep 10;196(12):1068–79. doi: 10.1007/s00066-020-01681-1 (PMC7483062; doi:10.1007/s00066-020-01681-1)
Supplement: Supplementary file 1 — ESM1: Questionnaire on the situation of radiotherapy in Germany during the SARS-CoV-2 pandemic [file 66_2020_1681_MOESM1_ESM.pdf]

DEGRO DEUTSCHE GESELLSCHAFT FÜR RADIOONKOLOGIE E.V.

Fragebogen zur Situation der Strahlentherapien in Deutschland während der SARS-CoV-2-Pandemie

*** 1. In welchem Land sind Sie tätig?**

- [ ] Deutschland
- [ ] Österreich
- [ ] Schweiz

*** 2. In welcher Einrichtung sind Sie tätig?**

- [ ] Einzel- oder Gemeinschaftspraxis
- [ ] Medizinisches Versorgungszentrum (MVZ)/ Primärversorgungszentrum (PVZ)
- [ ] Nicht-universitäre Klinik/Spital
- [ ] Universitätsklinik/-spital

3. Falls in Deutschland, geben Sie bitte an, in welchem Bundesland Ihre Institution liegt

*** 4. Haben Sie seit Beginn der SARS-CoV-2-Pandemie bereits Patienten gehabt, die während der Bestrahlungsserie eine nachgewiesene COVID-19-Erkrankung hatten?**

- [ ] Ja
- [ ] Nein

5. Falls ja, wie viele nachgewiesenen COVID-19-Fälle haben Sie gehabt?

6. Falls ja, wie sind Sie mit diesen Patienten umgegangen?

	Bestrahlung komplett abgebrochen	Bestrahlung >1 Woche unterbrochen	Bestrahlung weitergeführt bzw. <1 Woche unterbrochen	Anderes Vorgehen
Asymptomatische oder gering symptomatische Patienten				
Stärker symptomatische Patienten				

Kommentar

*** 7. Wie viele Patienten befinden sich derzeit bei Ihnen in einer Bestrahlungsserie (Pro Tag)?**

*** 8. Wie viele Patienten befanden sich kurz vor Beginn der SARS-CoV-2-Pandemie (Anfang 2/2020) in der Bestrahlungsserie (pro Tag)?**

*** 9. Führen Sie für die Patienten in der Bestrahlungsserie ein COVID-19-Screening (Screening irgendeiner Art) durch?**

- ☐ **Ja**
- ☐ **Nein**

10. Falls ja, wie oft pro Woche untersuchen Sie?

Befragung nach typischen Symptomen	
Temperaturmessungen	
pO2-Sättigungsmessungen	
Pulsmessungen	
Rachenabstriche und/oder nasaler Abstrich	

* 11. Gab es seit Beginn der SARS-CoV-2-Pandemie Mitarbeiter in der Radioonkologie, die positiv auf COVID-19 getestet wurden?

- [ ] Ja
- [ ] Nein

12. Falls ja, geben Sie bitte die Anzahl der Fälle und die Gesamtanzahl der Mitarbeiter an

	Anzahl der positiv Getesteten	Gesamtzahl
Ärzte		
MTRA		
Medizinphysiker		
Pflegekräfte		
Administration		

*** 13. Werden/wurden in Ihrer Einrichtung Antikörpertests durchgeführt, um eine durchgemachte Infektion zu erkennen und so Mitarbeiter gezielt einsetzen zu können?**

☐ Ja

☐ Nein

*** 14. Haben Sie Ihre Mitarbeiter in Gruppen eingeteilt?**

	Ja/Nein
Ärzte	
MTRA	
Medizinphysiker	
Pflegekräfte	
Sonstige	

Kommentar

*** 15. Erfolgt(e) eine Anpassung des Dienstplans im Rahmen der COVID-19-Pandemie?**

	Ja/Nein
Homeoffice für Ärzte	
Homeoffice für MTRA	
Homeoffice für Medizinphysiker	
Homeoffice für Pflegekräfte	
Homeoffice für Mitarbeitern mit Risikofaktoren (Alter, Lungenvorerkrankungen, Herz-Kreislauferkrankungen	

*** 16. Für wen besteht innerhalb der Radioonkologie die Pflicht Masken zu tragen (ohne Symptome bzw. ohne nachgewiesener SARS-CoV-2-Infektion)?**

	Standard Mund-Nasen-Schutz	FFP2/3 Maske
Patienten		
Ärzte		
MTRA		
Medizinphysiker		
Pflegekräfte		
Sonstige		

Kommentar

*** 17. Haben Sie Masken in ausreichender Menge zur Verfügung?**

	Keine Probleme	Teilweise Probleme	Starke Probleme
Standard Mund-Nasen-Schutz			

*** 18. Führen Sie für Ihre Mitarbeiter ein COVID-19-Screening durch?**

- [ ] Ja
- [ ] Nein

19. Falls ja, wie viele Mitarbeiter befragen / untersuchen Sie?

	Anzahl pro Woche
Befragung nach typischen Symptomen	
Temperaturmessungen	
pO2-Sättigungsmessungen	
Pulsmessungen	
Rachenabstriche	

Anderes Vorgehen

* 20. Weichen Sie von Ihrem bisherigen Standard der Strahlentherapie bei **kurativen** Konzepten wegen der SARS-CoV-2-Pandemie ab?

	Geschätzt in %
Unveränderte Standardfraktionierung	
Durch moderate Hypofraktionierung	
Durch starke Hypofraktionierung	
Verschiebung der Strahlentherapie	
Verzicht auf Strahlentherapie	

Kommentar

* 21. Weichen Sie von Ihrem bisherigen Standard der Strahlentherapie bei **palliativen** Konzepten wegen der SARS-CoV-2-Pandemie ab?

	Geschätzt in %
Unveränderte Fraktionierung	
Durch moderat stärkere Hypofraktionierung	
Durch sehr starke Hypofraktionierung/ Einzeit-RT	
Verschiebung der Strahlentherapie	
Verzicht auf Strahlentherapie	

* 22. Weichen Sie von Ihrem bisherigen Standard der Strahlentherapie bei **Entzündungsbestrahlungen** wegen der SARS-CoV-2-Pandemie ab?

	Geschätzt in %
Wird weiter zeitnahe durchgeführt	
Verschiebung der Strahlentherapie	
Verzicht auf Strahlentherapie	

Kommentar

* 23. Wie gehen Sie mit zusätzlichen medikamentösen Tumortherapien parallel zur Bestrahlung um (geschätzt in %)?

	Unverändert appliziert	Modifiziert appliziert	Abgebrochen/nicht appliziert
Chemotherapien			
Andere medikamentösen Tumortherapien			

Kommentar

* 24. Wie gehen Sie mit Nachsorgen um?

	Geschätzt in %
Routinenachsorgen werden regulär durchgeführt	
Routinenachsorgen werden verschoben	
Telefonische Nachsorge	
Videounterstützte Nachsorge	

Kommentar

25. Wollen Sie uns noch etwas mitteilen?
